# Assessment of Extremely Cold Subarctic Climate Environment Destruction of the Basalt Fiber Reinforced Epoxy (BFRE) Rebar Using Its Moisture Uptake Kinetics

**DOI:** 10.3390/polym13244325

**Published:** 2021-12-10

**Authors:** Anatoly K. Kychkin, Anna A. Gavrilieva, Alina A. Vasilieva, Aisen A. Kychkin, Mikhail P. Lebedev, Anastasia V. Sivtseva

**Affiliations:** 1 V.P. Larionov Institute of Physical and Technical Problems of the North Siberian Branch Russian Academy of Sciences, 1 Oktyabrskaya Street, Yakutsk 677980, Russia; gav-ann@yandex.ru (A.A.G.); sianva@yandex.ru (A.V.S.); 2 Federal Research Center, The Yakut Scientific Centre of the Siberian Branch of the Russian Academy of Sciences, 2 Petrovskogo Str., Yakutsk 677000, Russia; kiir@mail.ru (A.A.V.); icen.kychkin@mail.ru (A.A.K.); m.p.lebedev@mail.ru (M.P.L.)

**Keywords:** destruction, cold climate, epoxy binder, basalt fiber, BFRP, rebar, moisture uptake, diffusion coefficient, Fick’s diffusion, PCM

## Abstract

A quite simple method is proposed for the assessment of extremely cold subarctic climate environment destruction of the basalt fiber reinforced epoxy (BFRE) rebar. The method involves the comparison of experimentally obtained long-term moisture uptake kinetic curves of unexposed and exposed BFRP rebars. A moisture uptake test was carried out at the temperature of 60 °C and relative humidity of 98 ± 2% for 306 days. The plasticization can be neglected because of low-level moisture saturation (<0.41% wt.); the swelling and structural relaxation of the polymer network can be neglected due to the high fiber content of BFRP rebar; moisture diffusion into the basalt fibers can be neglected since it is a much lesser amount than in the epoxy binder. These assumptions made it possible to build a three-stage diffusion model. It is observed that an increase in the density of defects with an increase in the diameter of the BFRP rebar is the result of the technology of manufacturing a periodic profile. The diffusion coefficient of the BFRP rebar with a 6, 10, or 18 mm diameter increased at an average of 82.7%, 56.7%, and 30%, respectively, after exposure to the climate of Yakutsk during 28 months, whereas it was known that the strength indicators had been increased.

## 1. Introduction

The subject of the study is inextricably linked with the actual problem of ensuring high resistance of the strength properties of polymer composites to aggressive environments [1,2,3,4,5,6,7,8,9,10], including the forceful impacts of subarctic climate on the composites [10,11,12]. In this area, examples have been identified [2,13,14], showing that some mechanical indicators of polymer composite materials after an open-air exposure in cold and extremely cold subarctic climate deteriorated comparably or even more significantly than in warm-summer humid continental climate. In [12], it is shown that the indicators’ strength of basalt fiber reinforced polymer (BFRP) rebar, which has been under the influence of destructive processes of extremely cold subarctic climate for several years, has improved. Thus, it is necessary to find and confirm more sensitive indicators to the initial destruction of the BFRP rebar.

It was found that for carbon fiber reinforced plastic, fiberglass, and other plastics with an epoxy binder, one of such promising indicators is the diffusion coefficient during desorption of moisture [15,16,17]. In this case, desorption was carried out after the first equilibrium moisture content of the plastic. Moreover, only the binder’s abnormal effects of moisture absorption (plasticization, swelling, hydrolysis, additional hardening, structural relaxation, pore formation, microcracking) were taken into account. However, usually the initial free volume content is unknown; polymer composite materials (PCM) can contain defects and capillaries in the interface fiber/binder, depending on the initial composition, reinforcement design, manufacturing technology, and environmental conditions. This type of free volume can be the generating part of the moisture content of the PCM [18,19,20,21]. For a unidirectional PCM, the moisture uptake by capillaries can be distinguished by considering the moisture uptake by the samples of different heights. Thus, for an adequate quantitative evaluation of the free volume in unidirectional PCM, it is necessary that the samples with different heights were exposed to moisture for a long time.

Thus, this work proposes a quite simple method for evaluating extremely cold subarctic climate environment destructive processes of a basalt fiber reinforced polymer rebar. The way compares the experimentally obtained long-term moisture uptake kinetic of unexposed BFRP rebar with the kinetic of open-air exposed BFRP rebar. A constructive comparison should be achieved by introducing the minimum number of parameters needed to approximate the BFRP rebar moisture uptake. It should be noted that the quantitative control of free volume in a BFRP rebar forms the basis for reliable prediction of the rebar mechanical properties in the long term [19,22].

## 2. Materials and Methods

### 2.1. Raw Materials

The reinforcing member was RBN 13-2400-4S basalt roving manufactured by “TBM” LLC (Yakutsk, Russia) in Yakutsk (fiber diameter of 13 microns). The nominal linear density of roving was 2400 tex. Breaking load of roving was not less than 320 mN/tex. The binder was an epoxy resin (ED-22) (Kukdo chemical Co.LTD, Seoul, Korea), which was “hot” cured with a suitable amount of hardener in the presence of accelerator. The hardener isomethyltetrahydrophthalic anhydride (iso-MTHPA) (JSC Sterlitamak Petrochemical Plant, Sterlitamak, Russia) was chosen because the systems cured with it have high mechanical properties, excellent waterproof, good electrical properties, and resistance to climatic impact. 2,4,6-tris(dimethylaminomethyl)phenol (UP 606/2) accelerator was chosen due to its high catalytic activity.

### 2.2. Manufacture of Composite and Specimen Preparation

The BFRP rebar was manufactured following Technical Specifications 2296-001-86166796-2013 “Non-metallic composite armature made from basalt plastic.” BFRP rebars were prepared using the pultrusion molding process. Continuous basalt rovings are impregnated with an epoxy binder; some of the impregnated rovings were pulled through the central spinneret molding an inner rod, then this rod was cured (8 kgF); the remainder of the impregnated rovings are pulled through the peripheral spinneret molding an outer layer and fixed to the cured inner rod. Finally, the outer layer is wrapped by polyamide thread, molding a periodic profile of the rebar.

**Figure 1:**
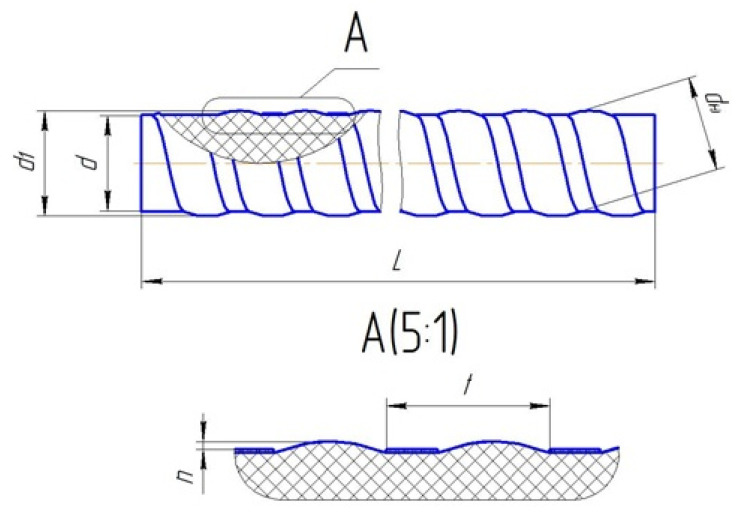
The keys of periodic rebar profile.

The profile and key of the size of rebar components are shown in Figure 1. The rebar with a diameter of d means that a diameter of the inner rod is d, above which a step of l wavy protrusions of length n rise, forming the outer diameter of d1 and the polyamide thread with a length of dн (Table 1). The object of research is the BFRP rebar with diameters of 6, 10, and 18 mm. Test specimens of 5, 10, 30, 50, 70, and 100 mm height were cut, and cross-sections were polished from each BFRP rebar.

### 2.3. Climatic Aging of BFRP Rebar

**Figure 2:**
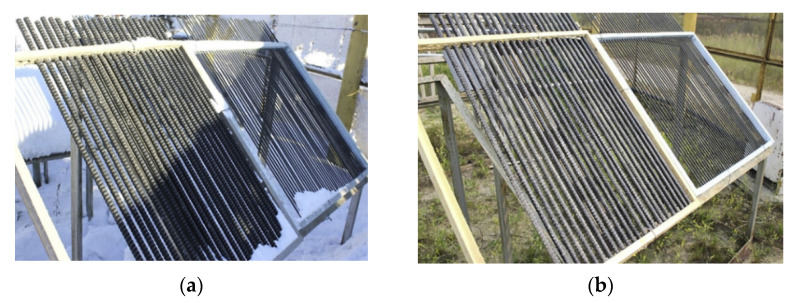
Open stands with BFRP rebar in Yakutsk in winter (**a**), and in summer (**b**).

The FBRP rebars were exposed to the extremely cold subarctic climate in Yakutsk for 28 months (Figure 2). The minimum temperature was registered as minus 64.4 °C. Per year the maximum temperature difference reaches 100 °C, average wind speed is 1.8 m/s, average relative humidity is 68%, 237 mm of precipitation falls, the solar radiation is 3680 MJ/m^2^.

### 2.4. Moister Absorption Tests and Kinetic Moisture Uptake Profile

The test method for moisture absorption of BFRP rebar was constituted with the recommendations of ASTM [23]. The test and preconditioning chamber was a Binder ED53 oven (Germany) over silica gel(BINDER GmbH, Tuttlingen, Germany). Preconditioning of the sample was for 14 days prior to its constant weight. The test relative humidity was 98 ± 2%. The samples were placed over distilled water in a desiccator 2-240 TY 25.11.1024-88 (JSC “Khimreaktivsnab”, UFA, Russia). The measurement time interval was one day. The test balance was an Ohaus analytical balance (Ohaus, NJ, USA) with measurement error 0.1 mg and range 0.01–210 g, accuracy class I. The percent change in mass determined the kinetic moisture uptake profile of the sample

(1) $$M_{t}\left(\%\right)=\frac{M_{t}}{M_{0}}\cdot 100\%=\frac{M-M_{0}}{M_{0}}\cdot 100\%,$$

where $M_{t}\left(\%\right)$ was the weight gain, $M_{t}$ was the amount of diffusing substance that got into the cylinder on time t, M was the weight of sample at time t, and $M_{0}$ was the initial sample weight after drying at t=0. Three samples were tested for each rebar mass, and the average moisture uptake data with standard deviation were reported.

### 2.5. Fickian Diffusion Model for a Long Cylinder

For a homogeneous continuous cylinder with radius R, in which diffusion through the butts can be neglected, the diffusion is described by the Fick equation [24]

$$\frac{\partial C}{\partial t}=\frac{1}{r}\frac{\partial }{\partial r}\left(rD\frac{\partial C}{\partial r}\right),$$

where C is a moisture concentration, r is a radial coordinate and D is the diffusion coefficient. Let the constant concentration be adhered to the cylinder boundary C(R,t)=const. There is no penetrator in the sample at the initial time C(R,0)=0 (0<r<R). If $M_{\infty }$ is the corresponding quantity after the infinite time (saturated or equilibrium), then according to [25]

(2) $$M_{t}=M_{\infty }\left(1-\frac{4}{R^{2}}\right)\sum_{n=1}^{\infty }\frac{\exp\left(-\alpha_{n}^{2}Dt\right)}{\alpha_{n}^{2}},$$

provided the $\alpha_{n}^{}$ are roots of $J_{0}\left(R\alpha_{n}^{}\right)=0$, where $J_{0}\left(r\right)$ is the Bessel function of the first kind of order zero.

### 2.6. Macroanalysis of Material Structure

Macroanalysis of samples was done using Nikon Eclips LV100 (Nilon, Tokyo, Japan) at magnifications of 30; 50; 100; and 500.

## 3. Results and Discussions

### 3.1. Experimental Moisture Uptake Kinetics (a Three-Stage Diffusion)

**Figure 3:**
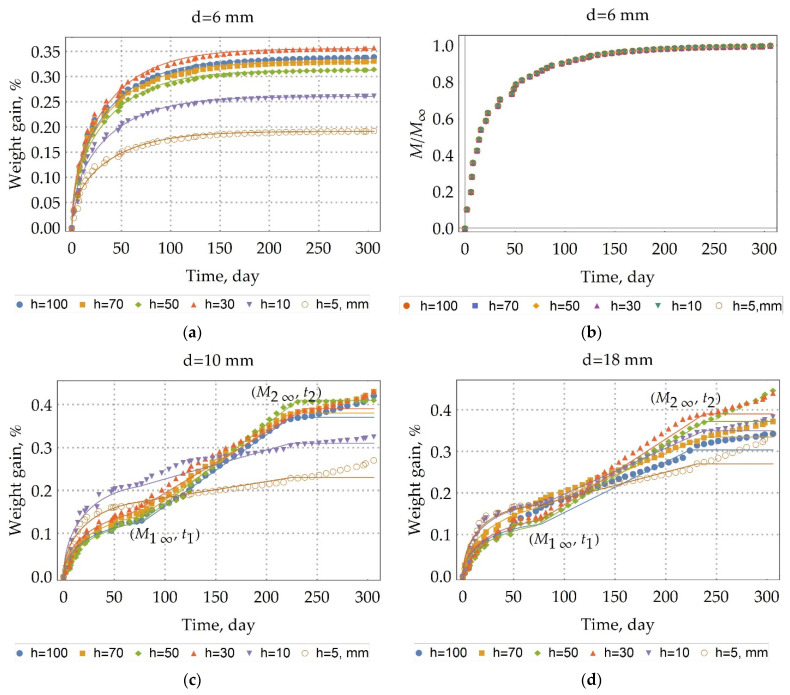
Moisture uptake kinetics (60 °C/98%RH) for the samples of unexposed BFRP rebars with a height (h, mm) and: (**a**) with a diameter of 6 mm and; (**b**) with a diameter of 6 mm related to $M_{\infty }$; (**c**) with a diameter of 10 mm; (**d**) with a diameter of 18 mm. The periodic profile of the rebars is a cause of the first and second stages of diffusion.

Moisture uptake kinetics were calculated as the percent change in mass (1). The moisture uptake kinetics are presented in Figure 3 for the rebars were stored in the warehouse (unexposed rebars). The kinetics are sorted by the diameter of the rebar. The results show differences in moisture uptake behavior between the rebar with a diameter of 6 mm and the rebar with a 10, 18 mm diameter. In contrast to the kinetics of moisture uptake in the rebars with a diameter of 6 mm, the kinetics of moisture uptake in the rebars with a 10, 18 mm diameter during the period of moisture exposure represents the circumstance of three-stage diffusion (see Figure 3c,d). At the first stage, there is a quasi-equilibrium $\left(M_{1\infty },t_{1}\right)$; at the second stage $\left(M_{2\infty },t_{2}\right)$, there is a linear increase in moisture absorption, and at the third stage, there is a sharp jump in sample mass or the unchanging of the sample mass. This observation of three-stage diffusion indicates that a time-variable diffusion model is most suitable for describing moisture diffusion of unexposed BFRP rebars with a 10 or 18 mm diameter [26]. However, this time-variable character is not suitable for a quick and adequate comparison of kinetic curves.

**Figure 4:**
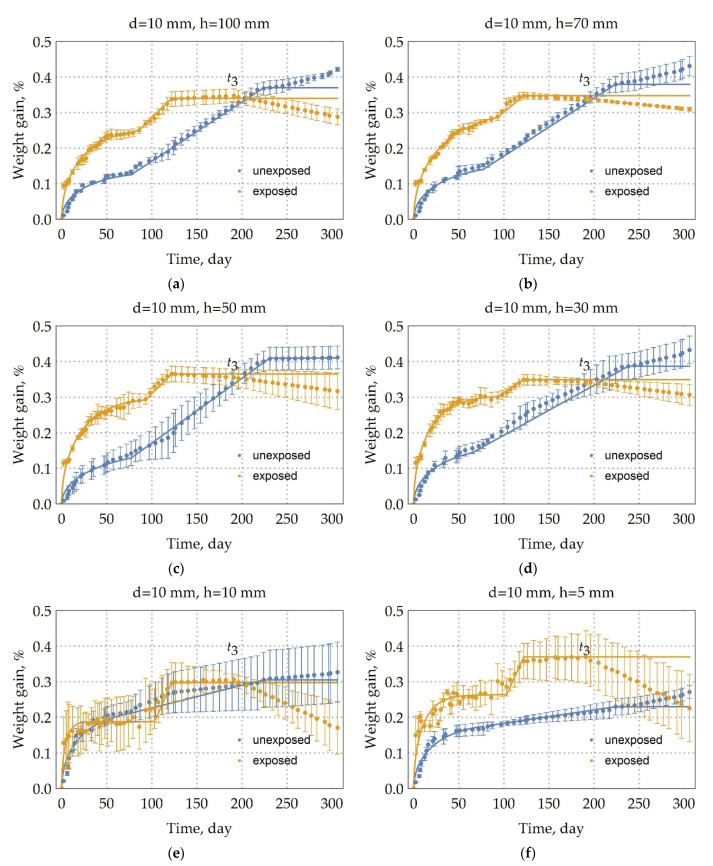
Moisture uptake kinetics (60 °C/98%RH) for the samples of unexposed BFRP rebars and the samples of exposed BFRP rebars in an extremely cold subarctic climate of Yakutsk for 28 months with height (h, mm) and with the diameter of 10 mm. (**a**) 100 mm height sample; (**b)** 70 mm height sample; (**c**) 50 mm height sample; (**d**) 30 mm height sample; (**e**) 10 mm height sample; (**f**) 5 mm height sample. Moisture uptake of the exposed rebars is the three-stage diffusion.

**Figure 5:**
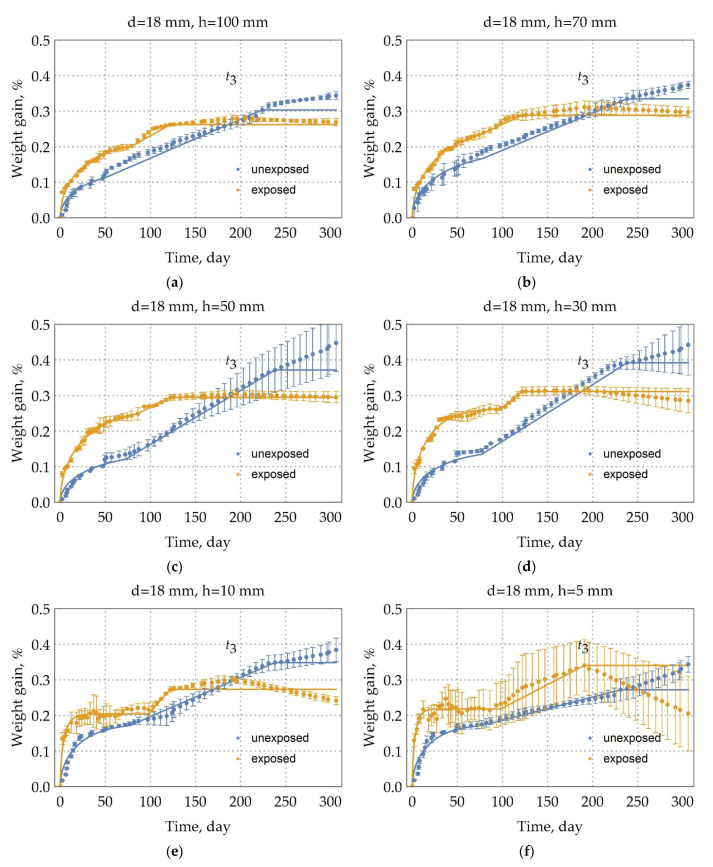
Moisture uptake kinetics (60 °C/98%RH) for the samples of unexposed BFRP rebars and the samples of exposed BFRP rebars in an extremely cold subarctic climate of Yakutsk for 28 months with height (h, mm) and with the diameter of 18 mm. (**a**) 100 mm height sample; (**b)** 70 mm height sample; (**c**) 50 mm height sample; (**d**) 30 mm height sample; (**e**) 10 mm height sample; (**f**) 5 mm height sample. Moisture uptake of the exposed rebars is the three-stage diffusion.

**Figure 6:**
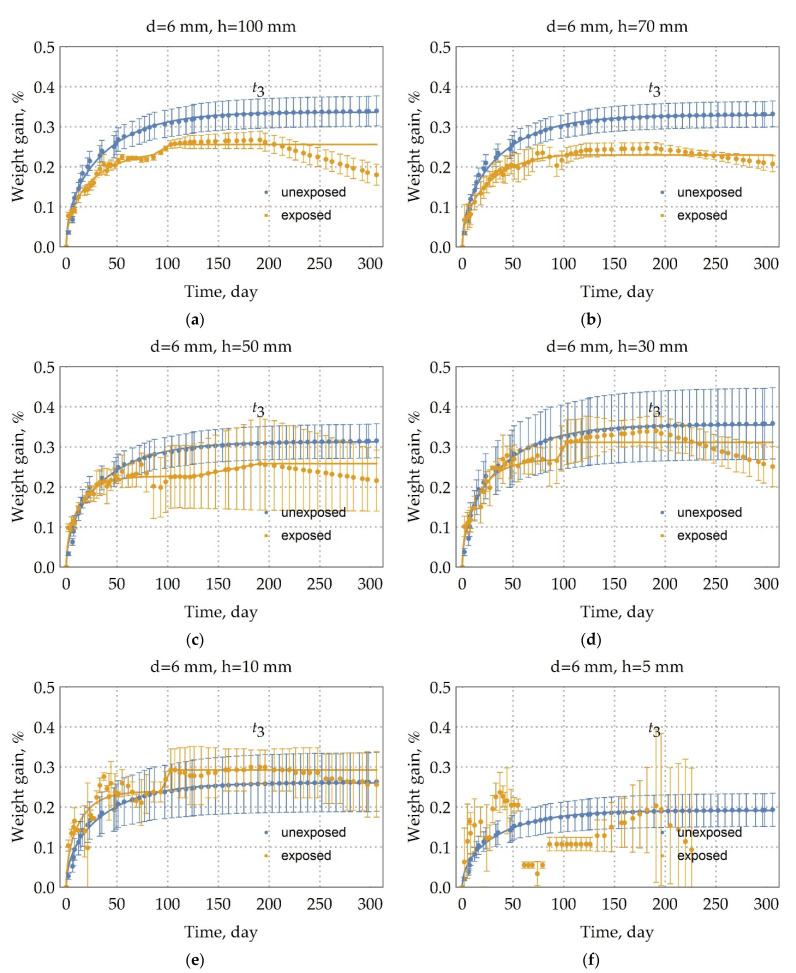
Moisture uptake kinetics (60 °C/98%RH) for the samples of unexposed BFRP rebars and the samples of exposed BFRP rebars in extremely cold subarctic climate of Yakutsk for 28 months with height (h, mm) and with the diameter of 6 mm. (**a**) 100 mm height sample; (**b**) 70 mm height sample; (**c**) 50 mm height sample; (**d**) 30 mm height sample; (**e**) 10 mm height sample; (**f**) 5 mm height sample Moisture uptake of the exposed rebars is the three-stage diffusion.

The moisture uptake kinetics are presented in Figure 4, Figure 5 and Figure 6 for the samples of the DFRP rebars that were exposed in an extremely cold subarctic climate of Yakutsk for 28 months. It can also be seen from these curves of kinetics that there is three-stage diffusion. At the first stage, there is a quasi-equilibrium; at the second stage, there is a linear increase in moisture absorption; and at the third stage, there is a linear decrease in mass. Additionally, this observation of three-stage diffusion indicates that a time-variable diffusion model is the most fitted for describing moisture diffusion in these exposed rebars [26]. However, this time-variable character is not suitable for a quick and adequate comparison of kinetic curves.

### 3.2. Approximation of Moisture Uptake Kinetics

As one could see from Figure 3b value $M/M_{\infty }$ does not depend on the height of the sample, so the Fickian model could predict the experimental behavior of the test unexposed d6 rebars within the test period very well. To determine approximation parameters: the diffusion coefficient D and the equilibrium moisture uptake $M_{\infty }$, the least-squares fitting technique was implemented. In this technique, the sum of the square of the offsets of the experimental weight gain from the calculated one

$${\left(\Delta M\right)}^{2}\equiv \sum_{n=1}^{N}{\left(M_{t,n}^{\exp}\left(\%\right)-M_{t,n}^{cal}\left(\%\right)\right)}^{2}\to \min,$$

was minimized by approximation parameters were varied, using for Fickian model, $M_{t,n}^{\exp}$ was the nth experimental data of the weight gain at the time t, $M_{t,n}^{cal}\left(\%\right)$ was determined by Equation (2). Here, N is the aggregate number of the experimental points. The approximated D and $M_{\infty }$(%) are taken as the diffusion coefficient and the saturated moisture content (%) of a sample and listed in Table 2. Additionally, listed in Table 2 are the coefficient of determination (R2) values:

(3) $$R2=1-\frac{{\left(\Delta M\right)}^{2}}{\sum_{n=1}^{N}{\left(M_{t,n}^{\exp}\right)}^{2}-\sum_{n=1}^{N}M_{t,n}^{\exp}/N},$$

From Equation (3), we assess the proportion of the experimental data described by the approximation. If R2=1 in Equation (3), the model is a complete description of the experimental data. The values D, $M_{\infty }$(%) and R2 were calculated using Wolfram Cloud Basic (Wolfram Research, Illinois, United States). The calculated values of the coefficient of determination (in Table 2) confirm our suggestion that the Fick model describes moisture diffusion in the unexposed BFRP rebars with a diameter of 6 mm quite well conditions.

**Figure 7:**
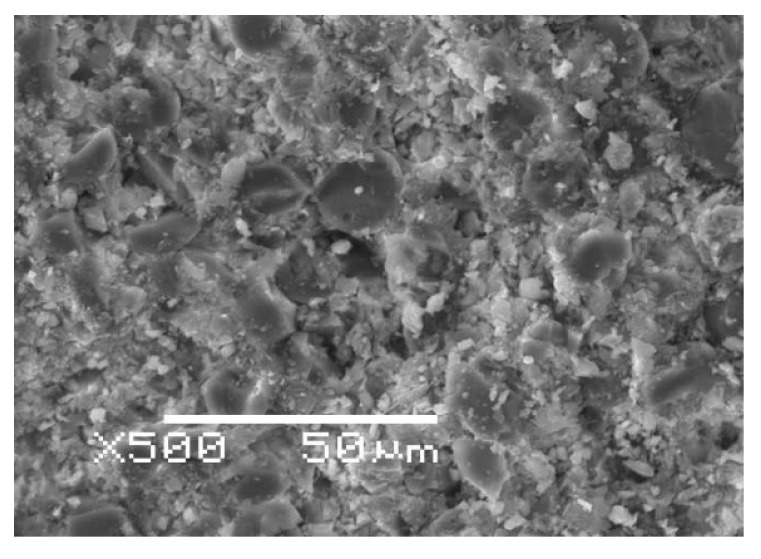
The cross-section of the BFRP rebar. A high degree of fiber filling can be observed.

Furthermore, an analysis of the rebar structure showed that rovings are distributed evenly over its cross section, and the degree of fiber filling is high, about 79% wt. (see Table 1), as illustrated in Figure 7. An increase in sample volume due to swelling and structural relaxation of the polymer network can be neglected due to high fiber content, since the resilience of thin basalt fibers is included in counteracting the swelling and structural relaxation. Thus, the first two stages of uptake are not explained by the typical two-stage Flory [27], Bagley and Long [28], and Newns [29] for glassy polymer systems and other theories arising from them. Moreover, this is not observed in the rebar with a diameter of 6 mm (see Figure 3a). The moisture uptake by a basalt fiber can be neglected since the moisture saturation content of the basalt fiber is much less than the moisture saturation content of the epoxy binder.

**Figure 8:**
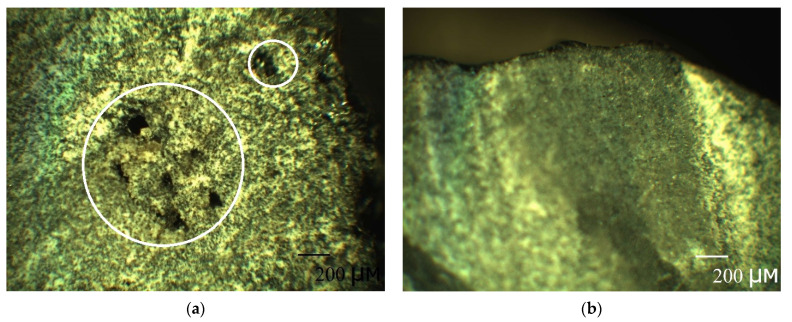
Pictures of the cross-section of unexposed BFRP rebar with a diameter: (**a**) 18 mm and (**b**) 8 mm exposed sample. With an increase in the rebar diameter defects can be observed.

The mechanism of the second stage can be clearly distinguished by considering the change in the kinetic curves with a height and a diameter in Figure 3c,d. It is known that microchannels occur mainly at fiber/matrix interfaces. The outer layer of the rebar is adjusted to the inner one to create a periodic profile (see Section 2.2. Composite specimen preparation and characterization), creating defects and microchannels. Clearly, with an increase in height, the volume and density of such defects and microchannels increase. In Figure 3c there is the increase of gain $\left(M_{2\infty }-M_{1\infty }\right)$ with a height increase. The pre-existing quasi-equilibrium suggests that there is some barrier. After overcoming the barrier, the second stage begins. The second stage is linear. The second stage for h = 50 mm sample ends with a complete stop of weight gain. Figure 3d shows the same behavior, but the second stage is slightly smeared because, with an increase in diameter, the volume and density of defects increase according to a periodic profile’s manufacture (Figure 8). All this fact suggests that, at first, the rebar’s mesopores, micropores, and microchannels are filled by a simple diffusion mechanism through polymer nanopores (the first stage), and then they are filled by capillary condensation of moisture (the second stage). The simple diffusion mechanism of the first stage suggests that the Fick model can be applied (2). Moreover, according to the current view [30], for all fiber-reinforced polymer composites, the first phase of moisture uptake proceeds by simple diffusion according to the Fickian model.

The mechanism of the third stage can be explained by hydrolysis of the epoxy binder. The moisture breaks a polymeric chain under long-term exposure to thermal and humidity. Two phenomena happen simultaneously: (a) the weight gain due to water uptake in the voids constructed by this degradation and cracking of the binder, and (b) the weight loss due to hydrolysis and subsequent binder spalling [31,32]. The different rates of weight gain and weight jump in the third stage suggest the predominance of the mechanism (a) for the unexposed BFRP rebar with a diameter of 10, 18 mm (Figure 3b,c).

Thus, we approximate the first stage by the Fick model $\left(M_{1\infty }(\%),D\right)$ (2), the second stage by linear growth from point $\left(M_{1\infty }(\%),t_{1}\right)$ to point $\left(M_{2\infty }(\%),t_{2}\right)$. The value $M_{2\infty }$ will be considered as the moisture saturation content, which is responsible for the free volume in the rebar, since the third stage describes the degradation of rebar because of exposure to moisture. The modulated parameters and the adequacy of the three-stage diffusion model of the unexposed BFRP rebar are shown in Table 2.

In Figure 3, the approximation of the experimental data by three-stage diffusion (Table 2) is indicated by solid lines. The analysis of Table 2 shows that the model we constructed adequately describes moisture absorption by the unexposed BFRP rebar; the value of a coefficient of determination (3) is not less than 0.93. It takes 77 days to start the second stage. The maximum moisture content is 0.41%. The low value of the moisture content indicates that the plasticization can be neglected. The average weight gain of long (h = 30, 50, 70, 100 mm) rebar with a 10 mm diameter is 0.23%, with an 18 mm diameter is 0.20% at the second stage. Moreover, the second stage with a 6 mm diameter is absent. According to the mechanism of the second stage, it can be suggested that the unexposed BFRP rebar with a 6 mm diameter has an excellent adhesion between the fiber and the binder, the density of the microchannels is higher in the unexposed BFRP rebar with a 10 mm diameter than in unexposed BFRP rebar with an 18 mm diameter (see Figure 3). An increase in the quasi-equilibrium content of moisture with a decrease of height for rebar with a 10 mm diameter from 0.14% to 0.22%, for rebar with an 18 mm diameter from 0.13% to 0.18%, and a decrease in the quasi-equilibrium content of moisture from 0.34% to 0.19% for a 6 mm diameter rebar can be explained by the predominance of one of the following phenomena: (a) with a decrease in the sample height, the density of defects in rebar fabrication decreases, and (b) with an increase in the diameter of the rebar, it is a more significant fracture when cutting the specimen.

### 3.3. Evaluation of the BFRP Rebar Destruction

The modulated parameters by three-stage diffusion and the assessment of model adequacy for BFRP rebar (see subparagraph 3.2), exposed in an extremely cold subarctic climate during 28 months, are shown in Table 3. In Figure 4, Figure 5 and Figure 6, there are solid lines that correspond to this approximation. The three-stage diffusion adequately describes moisture absorption by the unexposed BFRP rebar; the value of a coefficient of determination (3) is not less than 0.91 for a long sample.

**Figure 9:**
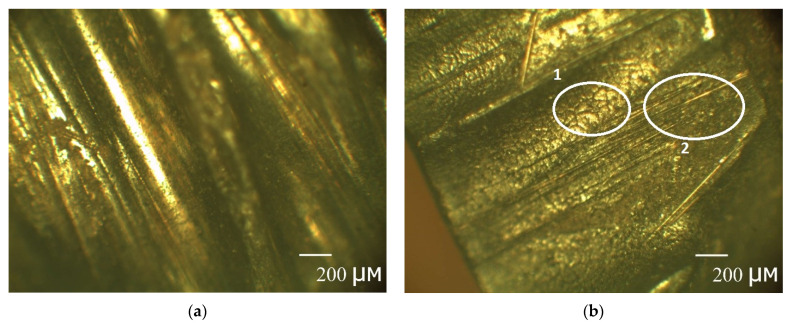
Pictures of the surface of the BFRP rebar: (**a**) unexposed sample, and (**b**) exposed sample. 1—the cracking of the epoxy binder and 2—the fiber denudation can be observed.

The study [33] dealt with the destruction of an epoxy binder on the surface of basalt textolite under the influence of the same cold climate for two years: in the form of cracking up to 1 micron in-depth, bare fibers and single depressions no more than 30 microns. Similar destruction can be observed for the studied case of the BFRP rebar in Figure 9.

It is expected that such cracking of the epoxy binder on the surface of the exposed rebar will increase the surface area of moisture penetration; thus, moisture saturation of the exposed sample will occur faster than the unexposed sample. Indeed, this can be seen in Figure 5 and Figure 6. The diffusion coefficient of the long sample of exposed rebar with a 6, 10, and 18 mm diameter increased at an average of 82.7%, 56.7%, and 30%, respectively (See Table 3). It is clear that this difference is explained by the same thickness of the surface destruction. The change in the diffusion coefficients of samples with a height of 10 and 5 mm was not taken into account, since here the influence of the edge of sample prevails.

The first stage of studying exposed rebar ran mainly 93 days. As can be seen from Figure 4, Figure 5 and Figure 6 and the analysis of Table 3, the moisture saturation content of the exposed samples is less than that of the unexposed samples by a maximum of 20 percent. The average weight increase at the second stage of long rebar with a 10 mm diameter is 0.06%, 18 mm diameter is 0.04%, and 6 mm diameter is 0.02%. The existence of a linear second stage of diffusion in exposed rebar with 6 mm diameter, in contrast to diffusion in unexposed rebar with 6 mm diameter, suggests that microchannels occur in the BFRP rebar due to the influence of the climate of Yakutsk.

Analysis of Table 3 and the kinetics of moisture absorption in Figure 4, Figure 5 and Figure 6 show that after exposure to the climate of Yakutsk for two years, the kinetics of moisture uptake is almost independent of the diameter of the sample. Thus, the difference in the kinetics of moisture uptake for unexposed rebar can be explained by the initial structural nonequilibrium. Structural disequilibrium depends on the mode of reinforcement formation and relaxes at the early stage of climatic aging [34].

**Figure 10:**
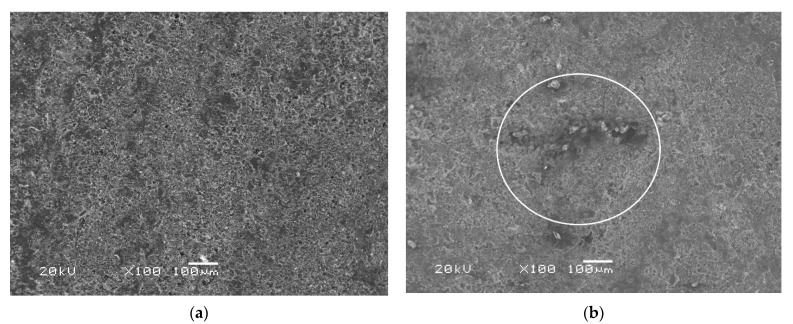
Pictures of the cross-section of the BFRP rebar: (**a**) unexposed, and (**b**) exposed sample. The subsequent epoxy binder spalling can be observed.

As seen from Figure 4, Figure 5 and Figure 6 and Figure 10, the third stage of the unexposed rebar begins after 191 days and suggests the mechanism’s predominance (b) weight loss due to hydrolysis and subsequent binder spalling.

## 4. Conclusions

Water saturation of PCM in comparison with moisture saturation has been extensively studied. It seems that water saturation is a well-known two-stage diffusion, while moisture saturation is three-stage diffusion. Until now, the mechanism of the second stage of moisture diffusion in PCM has no clear explanation. Apparently, in PCM with a high density of fiber filling, the first stage of moisture diffusion corresponds to Fick’s diffusion, the second stage is responsible for the capillary filling of defects and/or microchannels, and at the third stage of diffusion, the binder is hydrolyzed. According to this mechanism, it is possible to draw conclusions about the distribution of free volume in the composite by a quite simple but long-term method of moisture saturation of the composite without destroying its integrity. Thus, the change in the diffusion coefficients at the first stage will indeed reflect the degree of surface destruction of PCM with a high density of fiber filling, and the absence of the second stage of moisture saturation will reflect good adhesion between the fiber and the binder.

Moisture uptake test was carried out at the temperature of 60 °C and relative humidity of 98 ± 2% during 306 days. The three-stage diffusion model adequately describes moisture absorption by the BFRE rebar; a coefficient of determination is not less than 0.93. It takes 77 days to start the second stage. The three-stage diffusion model adequately describes moisture absorption by the BFRE rebar after exposure to an extremely cold subarctic climate of Yakutsk for 28 months; a coefficient of determination is not less than 0.85. It takes 93 days to start the second stage and 191 days to start the third stage. The formation of a periodic profile of the BFRP rebar leads to the fact that as the rebar’s diameter increases, there are defects in the periphery of the rebar, as well as microchannels in the fiber/binder interface. The maximum value of the moisture saturation content is 0.41%. The moisture saturation content of the exposed samples is less than that of the unexposed samples by a maximum of 20%. The diffusion coefficient of the long sample of the BFRP rebar with a 6, 10, and 18 mm diameter increased at an average of 82.7%, 56.7%, and 30%, respectively, after exposure to an extremely cold subarctic climate of Yakutsk for 28 months. A future research direction is to validity the second stage of diffusion in BFRP rebar.

## Figures and Tables

**Table 1:** Technological modes of forming a periodic profile of rebar.

d (mm)	d1 (mm)	dн (mm)	n (mm)	l (mm)	Fibres Mass Share (%)
6	7	6.5	0.5	5–7	77
10	12	10.5	1	7–9	78
18	23	18.4	1.5	8–12	70

**Table 2:** Adequacy and parameters of the three-stage diffusion model of unexposed BFRP rebar.

A Diameter	A Height, mm	$M_{1\infty }(\%)$	$D\cdot 10^{6},\;{\mathrm{cm}}^{2}/\mathrm{Day}$	$\frac{D}{R^{2}}\cdot 10^{3},\;1/\mathrm{Day}$	*t*_1_, Day	*t*_2_, day	$M_{2\infty }(\%{)}_{\;}$	*t*_3,_ day	*R*2 from Equation (3)
6 mm	100	0.34	3.49	3.88	-	-	-	-	0.94
	70	0.33	3.49	3.88	-	-	-	-	0.94
	50	0.31	3.49	3.88	-	-	-	-	0.94
	30	0.36	3.49	3.88	-	-	-	-	0.93
	10	0.26	3.49	3.88	-	-	-	-	0.95
	5	0.19	3.49	3.88	-	-	-	-	0.96
10 mm	100	0.14	11.87	4.75	77	224	0.37	224	0.95
	70	0.16	9.84	3.94	77	224	0.38	224	0.94
	50	0.16	7.20	2.88	77	231	0.41	231	0.95
	30	0.17	10.35	4.14	65	238	0.39	238	0.94
	10	0.22	14.47	5.79	72	224	0.31	224	0.93
	5	0.19	13.79	5.52	77	224	0.23	224	0.94
18 mm	100	0.13	40.66	5.02	65	224	0.30	224	0.95
	70	0.19	29.73	3.67	77	238	0.33	238	0.95
	50	0.14	30.12	3.72	77	238	0.37	238	0.94
	30	0.15	34.39	4.25	77	238	0.39	238	0.94
	10	0.18	50.08	6.18	77	238	0.35	238	0.94
	5	0.18	57.05	7.04	77	231	0.27	231	0.94

**Table 3:** Adequacy and parameters of the three-stage diffusion model of exposed BFRP rebar.

A Diameter	A Height, mm	$M_{\infty }(\%{)}_{\;}$ from Equation (2)	$\frac{D}{R^{2}}\cdot 10^{3},\;1/\mathrm{Day}\;$	$D\cdot 10^{6},\;{\mathrm{cm}}^{2}/\mathrm{Day}$	*t*_1,_ Day	*t*_2,_ Day	$M_{2\infty }(\%{)}_{\;}$	*t*_3,_ Day	*R*2 from Equation (3)
6 mm	100	0.24	5.72	5.15	85	103	0.26	191	0.95
	70	0.24	5.27	4.74	93	103	0.23	191	0.93
	50	0.23	10.27	9.24	126	191	0.26	191	0.94
	30	0.27	8.38	7.54	93	103	0.31	191	0.91
	10	0.24	10.31	9.28	93	103	0.29	182	0.85
	5	-	-	-	-	-	-	0	-
10 mm	100	0.26	5.74	14.35	85	122	0.34	191	0.96
	70	0.31	4.15	10.38	93	122	0.34	191	0.95
	50	0.30	6.21	15.53	93	122	0.37	191	0.95
	30	0.31	7.65	19.12	93	122	0.35	191	0.95
	10	0.19	29.87	74.68	103	122	0.30	191	0.91
	5	0.26	12.72	31.80	103	122	0.37	191	0.86
18 mm	100	0.22	4.98	40.35	49	122	0.26	191	0.97
	70	0.27	4.07	32.97	75	122	0.29	191	0.96
	50	0.27	4.90	39.72	86	122	0.29	-	0.97
	30	0.26	7.63	61.82	98	122	0.31	191	0.95
	10	0.20	28.69	232.35	98	122	0.27	191	0.94
	5	0.22	38.91	315.21	98	122	0.28	191	0.94

## Data Availability

The data presented in this study are available on request from the corresponding author.
